# The AUstralian‐CLimate and Health Attitudes, RIsk and Trust in the sYstem (AU‐CLARITY) Study: A National, Representative Survey

**DOI:** 10.5694/mja2.70293

**Published:** 2026-09-10

**Authors:** Jeffrey Braithwaite, Carolynn L. Smith, Yvonne A. Zurynski, Louise A. Ellis, Georgia Fisher, Kate Churruca, Lisa Pagano, Samantha Spanos, Janet C. Long, Christina Rojas, S. Bruce Dowton

**Affiliations:** ^1^ Centre for Healthcare Resilience and Implementation Science, Australian Institute of Health Innovation Macquarie University Sydney New South Wales Australia; ^2^ Observatory on the Future of Healthcare, Australian Institute of Health Innovation Macquarie University Sydney New South Wales Australia; ^3^ Office of the Vice‐Chancellor Macquarie University Sydney New South Wales Australia

**Keywords:** climate change, health systems, public health, public policy

## Abstract

**Objectives:**

Climate change, associated with heat spikes, floods, wildfires and changing patterns of vector‐borne diseases, is a major determinant of health, and health system functioning. Understanding public perceptions of climate‐related health risks and system preparedness can support equitable policy responses and effective health messaging.

**Study Type:**

Cross‐sectional.

**Setting:**

Online survey from 20 June to 27 August 2025.

**Participants:**

6030 adults, aged ≥ 18 years, broadly representative of the Australian population.

**Main Outcome Measures:**

The AUstralian‐CLimate and Health Attitudes, RIsk and Trust in the sYstem (AU‐CLARITY) survey collected data on demographics, health status, experiences of climate‐related events, and views on climate change and health system preparedness.

**Results:**

Respondents (65%, 3910/6030) were concerned about climate change, yet only 45% (2735/6030) perceived it as a serious risk to their own health. Personal risk perception declined with age (odds ratio [OR] = 0.98; 95% confidence interval [CI], 0.98–0.98) and was higher among females (OR = 1.28; 95% CI, 1.15–1.43), First Nations people (OR = 1.44; 95% CI, 1.22–1.71), multilingual individuals (OR = 1.44; 95% CI, 1.22–1.71) and those with higher education (OR = 1.28; 95% CI, 1.12–1.46). Personal experience of a climate‐related event increased perceived risk (OR = 1.97; 95% CI, 1.73–2.24). Regional and remote residents were less likely to perceive health risk (OR = 0.76; 95% CI, 0.65–0.89). A third (37%; 2209/6030) of respondents believed that the healthcare system was prepared for climate change. Being female (OR = 2.35; 95% CI, 2.10–2.65), having a chronic condition (OR = 1.47; 95% CI, 1.28–1.68) and older age (OR = 1.01; 95% CI, 1.01–1.01) increased perceptions that the system was unprepared. Those with higher education, who had been personally impacted, or identified as First Nations had more confidence in the system's preparedness.

**Conclusions:**

Many Australians recognise climate change as a health challenge; personal risk perception and confidence in system preparedness vary. Targeted health policies are needed to effectively communicate health risks and support healthcare's climate change resilience.

## Plain Language Summary

**The Known:** Climate change threatens human health and health system resilience. Demographic factors affect vulnerability to the impacts of climate change. Individuals tend to underestimate their personal risk from climate change.

**The New:** To our knowledge, this is the first survey to examine Australians' perceptions of health system preparedness for climate change. Most Australians have been affected by climate change. However, many are uncertain about the effects of climate‐related events on the health system and the system's readiness to respond.

**The Implications:** Public health messaging is needed to improve personal risk assessment and understanding of the effects of climate change on health systems.

## 1 Introduction

The health impacts of climate change in Australia are intensifying, with rising temperatures, extreme weather events, shifting patterns of vector‐borne diseases and air pollution increasingly contributing to morbidity and mortality [1]. Healthcare systems are vulnerable to climate‐shocks, and are critical in mitigating the population's risk of climate‐induced harms [1, 2, 3, 4].

Although individuals in scientific and policy fields recognise these challenges, less is known about the general public's understanding of the effects of climate change on health and their perspectives on healthcare's preparedness for climate‐related events [2, 3, 4]. Previous research suggests that perceptions of personal risk of climate change impacts are influenced by demographic factors [5, 6, 7, 8, 9, 10]. Experiences of climate‐related events may also influence health risk perceptions [11]. However, individual risk perception does not always align with actual vulnerability [5, 11, 12], and misperceptions or misinformation about climate effects can reduce the effectiveness of health messaging [13, 14]. The aim of this research was to clarify how perceptions, experiences and demographic factors interact to inform public perceptions. Understanding this is critical for designing climate‐resilient health policy, crafting public messaging and ensuring equitable responses to climate change.

## 2 Methods

We used an anonymous online survey conducted with a representative sample of Australian adults. The results presented were reported against the relevant guidelines and checklists the Checklist for Reporting Results of Internet E‐Surveys (CHERRIES) (Table S1) and the Checklist for Reporting of Survey Studies (CROSS) (Table S2) [15, 16].

### 2.1 Participants and Recruitment

Adults (aged ≥ 18 years) were enrolled through a digital data collection company (Dynata) with > 400,000 registered panellists. The company recruited 6030 Australian adults to reach quotas for representation by age, gender and geographical location [17]. The sampling algorithm ensured demographic representation and random selection from the panellists. Participants enrolled via email, with opt‐in consent, from 20 June to 27 August 2025, receiving reward points (e.g., gift cards) for survey completion. Dynata, a first‐party data platform, uses multiple methods to verify respondent authenticity and prevent fraudulent participation, such as assigning a unique identifier to enable compensation and prohibit duplicate submissions, and tracking quality of responses across surveys. Deidentified responses were provided to the researchers. The response rate was not calculated as the survey was open to all Dynata panellists and responses were capped at required quotas.

### 2.2 Survey

We surveyed opinions and experiences of climate change, its effect on health, views on the readiness of the health system to respond to climate‐related emergencies and on mitigating health system emissions. This article reports on respondent demographics, health system usage, and views and experiences of climate change.

Survey items were purpose‐designed or drawn from published surveys (Supporting Information, Section 2) [8, 10, 11, 18]. Closed‐ (binary, Likert scale, multiple response) and open‐ended response options were used, together with adaptive questioning. The survey was pilot tested by 667 respondents for length and clarity.

### 2.3 Data Analysis

Postcode data were mapped to the Australian Statistical Geography Standard, which allocates remoteness to five classes. Categories were recoded as a dichotomous variable: metropolitan (major cities) and regional (inner and outer regional, remote and very remote) to retain statistical power in the analyses [18, 19]. Disease status was determined using the 12 chronic conditions commonly reported by the Australian Institute of Health and Welfare [20].

Descriptive statistics (frequencies, proportions) were calculated. Subgroup analyses were undertaken by age (years), gender (male/female [non‐binary or other were excluded]), identifying as Aboriginal and/or Torres Strait Islander (hereafter, First Nations), multilingualism (English‐only/multiple languages spoken at home), education (up to high school/university degree and above), region (metropolitan/regional) and chronic disease status (absence of chronic condition/≥ one condition). Regression analyses (generalised linear models or binary logistic regression as appropriate) measured relationships: between participant demographics and reports of satisfaction with the healthcare received (1 = ‘Very dissatisfied’/‘Somewhat dissatisfied’, 2 = ‘Neither satisfied nor dissatisfied’, 3 = ‘Somewhat satisfied’/‘Very satisfied’); perception of personal health risk posed by climate change (1 = ‘No threat at all’/‘Not very much threat’, 2 = ‘A fair amount of threat’/‘A great deal of threat’); and opinions on healthcare's readiness to respond to climate‐related emergencies (1 = ‘No/Unsure’, 2 = ‘Yes’). Tests for multicollinearity or for parallel lines were conducted. Linearity was confirmed for the continuous variable (age). Missing data were managed through listwise deletion. Interactions between independent variables were tested using forward likelihood ratio and are reported when they improved the model fit. Adjusted odds ratios (ORs) are reported. The effect of requiring support from healthcare due to a climate‐related event on perceptions of health system preparedness was analysed using a chi square test. A one‐sample chi square test was used to test willingness to pay more to strengthen health system preparedness. SPSS (version 30.0.0) was used for all analyses. A *p*‐value of < 0.05 was considered statistically significant.

### 2.4 Ethics Approval

Ethics approval was obtained from the Macquarie University Human Research Ethics Committee (#520251932663004).

## 3 Results

### 3.1 Demographic Profile

Adults (6030: 51% female) from all Australian states and territories participated (Table 1). The average respondent age was 46 years (range: 18–91 years), with 13% (762) identifying as First Nations. One in five respondents spoke a language other than English at home (1117, 19%). Forty‐seven per cent (2855) held a bachelor's degree or higher. Two thirds (4127, 68%) were in paid employment; 19% (1121) were retired. One or more chronic conditions were reported by 55% (3335), including mental health conditions (1317, 22%), back problems (1098, 18%), arthritis (922, 15%) and asthma (766, 13%) (Table S3). The sample was broadly representative of the Australian population, although older and with a higher proportion of First Nations' respondents compared with the general population (Table 1).

**TABLE 1:** Respondents' demographics.

Demographics	Respondents, *n* (%)	Australia (%)^a^
Gender		
Male	2937 (49)	50
Female	3076 (51)	50
Non‐binary	13 (0.2)	1
Did not say	4 (0.1)	Not applicable
State/territory		
New South Wales (NSW)	1945 (32)	31
Victoria (VIC)	1548 (26)	26
Queensland (QLD)	1182 (20)	21
Western Australia (WA)	556 (9)	11
South Australia (SA)	516 (9)	7
Tasmania (TAS)	140 (2)	2
Australian Capital Territory (ACT)	92 (2)	2
Northern Territory (NT)	51 (1)	1
Urban index		
Metropolitan	4178 (69)	67
Regional/Remote	1852 (31)	33
Age group (years)		
18–24	781 (13)	9
25–34	1082 (18)	15
35–44	1150 (19)	13
45–54	1085 (18)	12
55–64	907 (15)	12
65+	1025 (17)	17
Speaks a language other than English at home (Multilingual)	1117 (19)	23
Identifies as Aboriginal and/or Torres Strait Islander (First Nations)	762 (13)	4
Had at least one chronic condition	3335 (55)	50

^a^ Sources for Australia data: Australian Bureau of Statistics [17, 21, 22], Australian Commission on Safety and Quality in Health Care [23].

### 3.2 Health, Healthcare Utilisation and Satisfaction

Nearly 48% of respondents (2871) described their health as ‘excellent’ or ‘very good’, with 33% (1982) reporting ‘good’ health. Almost 97% (5843) had accessed healthcare in the past year; most frequently general practice (4197, 70%), pharmacy (3357, 56%) and dental care (2492, 41%) (Table S4). Satisfaction with healthcare was high among 5843 individuals who accessed it in the past year: 86% (5024) of respondents reported being ‘somewhat’ or ‘very satisfied’ with healthcare received; 6% (373) expressed dissatisfaction. An ordinal logistic calculation revealed significant demographic effects: males, English‐only speakers, individuals without chronic conditions, with higher education and older individuals had higher odds of being satisfied with their care (*χ* ^2^(7) = 87.98, *p* < 0.001; *n* = 5823). There was no significant effect of First Nations status or location (Table 2).

**TABLE 2:** Satisfaction with quality of care received in the last 12 months.

Ordinal logistic regression: (*χ* ^2^(7) = 87.98, *p* < 0.001) *n* = 5823				
Predictor (reference category)	Wald *χ* ^2^	Odds ratio	95% CI (lower–upper bounds)	*p*
Location				
(Metropolitan) Regional	0.17	1.04	0.88–1.22	0.679^a^
Gender				
(Male) Female	4.53	1.18	1.01–1.38	0.033
First Nations				
(No) Yes	0.13	1.04	0.83–1.31	0.715^a^
Multilingual				
(No) Yes	12.93	1.40	1.17–1.69	< 0.001
Education				
(High school or below) University degree or above	8.89	0.79	0.67–0.92	< 0.001
Has ≥ 1 chronic condition				
(No) Yes	17.04	1.34	1.19–1.63	< 0.001
Age (per year)	45.52	1.02	1.01–1.02	< 0.001

Abbreviation: CI, confidence interval.

^a^ Not significant at the *p* < 0.05 level.

### 3.3 Perceptions of the Causes of Climate Change and Its Effects on Health

**FIGURE 1:**
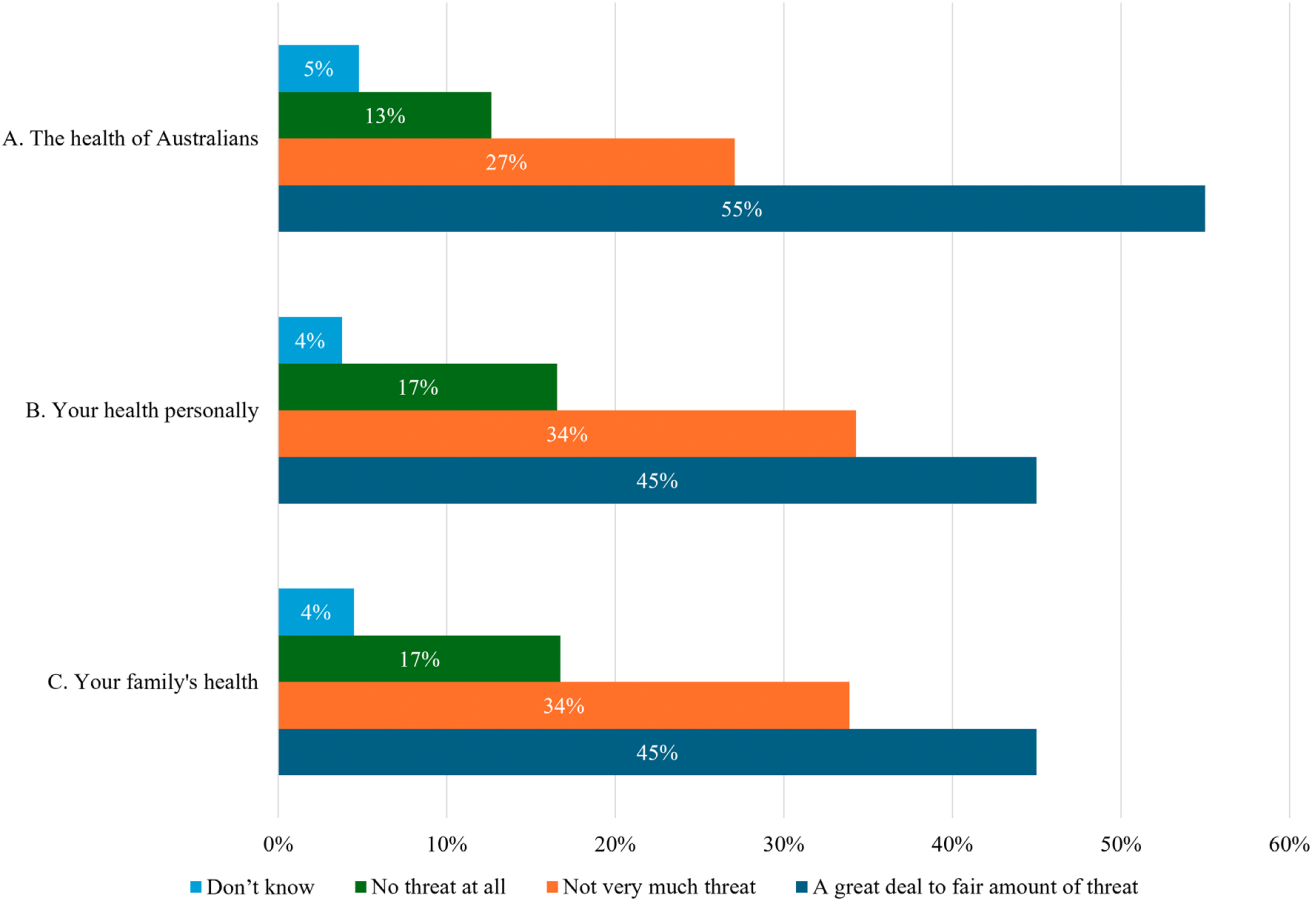
Participant concerns about the impact of climate change on the health of (A) Australians, (B) personal health and (C) their family's health. *N* = 6030 for each.

Most respondents (3910, 65%) were ‘fairly’ to ‘very concerned’ about climate change. The majority agreed that climate change was caused by human activity (4269, 71%), and was a global emergency (4159, 69%). Approximately 73% (4380) agreed that Australia was already feeling the effects of climate change; with over half believing that climate change was a threat to Australians' health (3344, 55%) (Figure 1a). Only 45% felt that climate change posed ‘a great deal’ or ‘a fair amount of threat’ to their personal (2735) or family's health (2705) (Figure 1b,c).

Sixty per cent (3632) of respondents had been personally affected by a climate‐related event, for example, bushfire, drought or flood, in the last 5 years. Twenty‐three per cent (821/3632) subsequently needed medical attention or psychological support.

### 3.4 Perceived Risk of Climate Change to Personal Health

A binary logistic regression revealed significant main effects of age, gender, location, education, First Nations status, language spoken at home and experiences of a climate‐related event on perceptions of personal health risks; with no significant effect of chronic condition (*χ* ^2^(8) = 551.30, *p* < 0.001; Nagelkerke *R* ^2^ = 0.12; *n* = 5780). The model fit was improved by using forward likelihood ratio to include interaction effects of language spoken × personal impact and location × education (*χ* ^2^(8) = 565.72, *p* < 0.001; Nagelkerke *R* ^2^ = 0.12; Hosmer–Lemeshow (HL) *p* = 0.20). Females (Exp(B) = 1.28; 95% CI, 1.15–1.43), individuals with higher education levels (Exp(B) = 1.28; 95% CI, 1.12–1.46), multilingual individuals (Exp(B) = 1.44; 95% CI, 1.22–1.71), First Nations respondents (Exp(B) = 1.44; 95% CI, 1.22–1.71) and individuals who had personal experience of a climate‐related event had higher odds of viewing climate change as a threat to their personal health (Exp(B) = 1.97; 95% CI, 1.73–2.24). Older (Exp(B) = 0.98; 95% CI, 0.98–0.98) and regional (Exp(B) = 0.76; 95% CI, 0.65–0.89) respondents were less likely to perceive climate change as a serious risk to their health.

The effect of personal impact on perceived risk to personal health was weaker, but still significant, for multilingual individuals (OR for personal impact: English‐only ≈1.91; multilingual ≈1.21). Higher education had a stronger effect on perceived threat in regional individuals (OR: Metropolitan ≈1.28; Regional ≈1.71) (Table 3).

**TABLE 3:** Perception of personal health risk from climate change.

Binary logistic regression: (*χ* ^2^(8) = 565.72, *p* < 0.001) *n* = 5780				
Predictor (reference category)	Wald *χ* ^2^	Odds ratio	95% CI (lower–upper bounds)	*p*
Location	11.27	0.76	0.65–0.89	< 0.001
(Metropolitan) Regional				
Gender	18.74	1.28	1.15–1.43	< 0.001
(Male) Female				
First Nations	17.90	1.44	1.22–1.71	< 0.001
(No) Yes				
Multilingual	27.95	1.91	1.50–2.43	< 0.001
(No) Yes				
Education	13.18	1.28	1.12–1.46	< 0.001
(High school or below) University degree or above				
Has ≥ 1 chronic condition	0.91	1.06	0.94–1.19	0.341^a^
(No) Yes				
Age (per year)	0.01	0.98	0.98–0.98	< 0.001
Personally impacted	106.37	1.97	1.73–2.24	< 0.001
(No) Yes				
Multilingual × Personally impacted	9.42	0.63	0.47–0.85	< 0.001
Location × Education	5.18	1.33	1.04–1.70	0.023

Abbreviation: CI, confidence interval.

^a^ Not significant at the *p* < 0.05 level.

### 3.5 Perceptions of Climate Change and the Australian Health System

Thirty‐nine per cent (2331) believed that the Australian health system is not being affected by climate change; 34% (2054) reported that it is; 27% (1645) did not know. Thirty‐seven per cent (2209) believed that the Australian health system was prepared to respond to climate‐related emergencies, compared with 34% (2069) who thought it was unprepared; 29% (1752) did not know. A binary logistic regression examining the effects of demographic factors on opinions about health system preparedness (‘No/Unsure’ vs. ‘Yes’) showed good fit (*χ* ^2^(11) = 956.55, *p* < 0.001; Nagelkerke *R* ^2^ = 0.20; HL *p* = 0.18; *n* = 6008). Inclusion of three interactions via forward stepwise likelihood ratio improved the model (*χ* ^2^(11) = 975.17, *p* < 0.001; Nagelkerke *R* ^2^ = 0.21; HL *p* = 0.62). Among main effects, being female (Exp(B) = 2.35; 95% CI, 2.10–2.65; *p* < 0.001), having a chronic condition (Exp(B) = 1.47; 95% CI, 1.28–1.68; *p* < 0.001) and older age (Exp(B) = 1.01; 95% CI, 1.01–1.01; *p* < 0.001) increased the likelihood of saying ‘No/Unsure’; while higher education (Exp(B) = 0.71; 95% CI, 0.63–0.80; *p* < 0.001), having been personally impacted by a climate‐related event (Exp(B) = 0.53; 95% CI, 0.46–0.62; *p* < 0.001) and identifying as First Nations (Exp(B) = 0.25; 95% CI, 0.20–0.30; *p* < 0.001) decreased the odds of responding ‘No/Unsure’. The main effects of location and language spoken at home were not significant.

On location × First Nations status: First Nations respondents residing in metropolitan areas had lower odds (OR = 0.25) of perceiving the system as unprepared (i.e., responding ‘No’ or ‘Unsure’) when compared with regional First Nations people (OR = 0.42). However, both groups were more likely to perceive the health system as prepared (i.e., respond ‘Yes’) than those not identifying as First Nations (Exp(B) = 1.70; 95% CI, 1.11–2.06; *p* = 0.14). Location × personally impacted by climate change revealed that being personally impacted by climate change had a smaller effect on the odds of saying the health system was prepared (‘Yes’) for regional residents compared with metropolitan (Exp(B) = 1.38; 95% CI, 1.06–1.79; *p* = 0.017); and chronic condition × language spoken at home showed that having a chronic condition increased the likelihood of saying ‘No/Unsure’ less among multilingual speakers than English‐only speakers (Exp(B) = 0.72, 95% CI, 0.54–0.96, *p* = 0.024).

**FIGURE 2:**
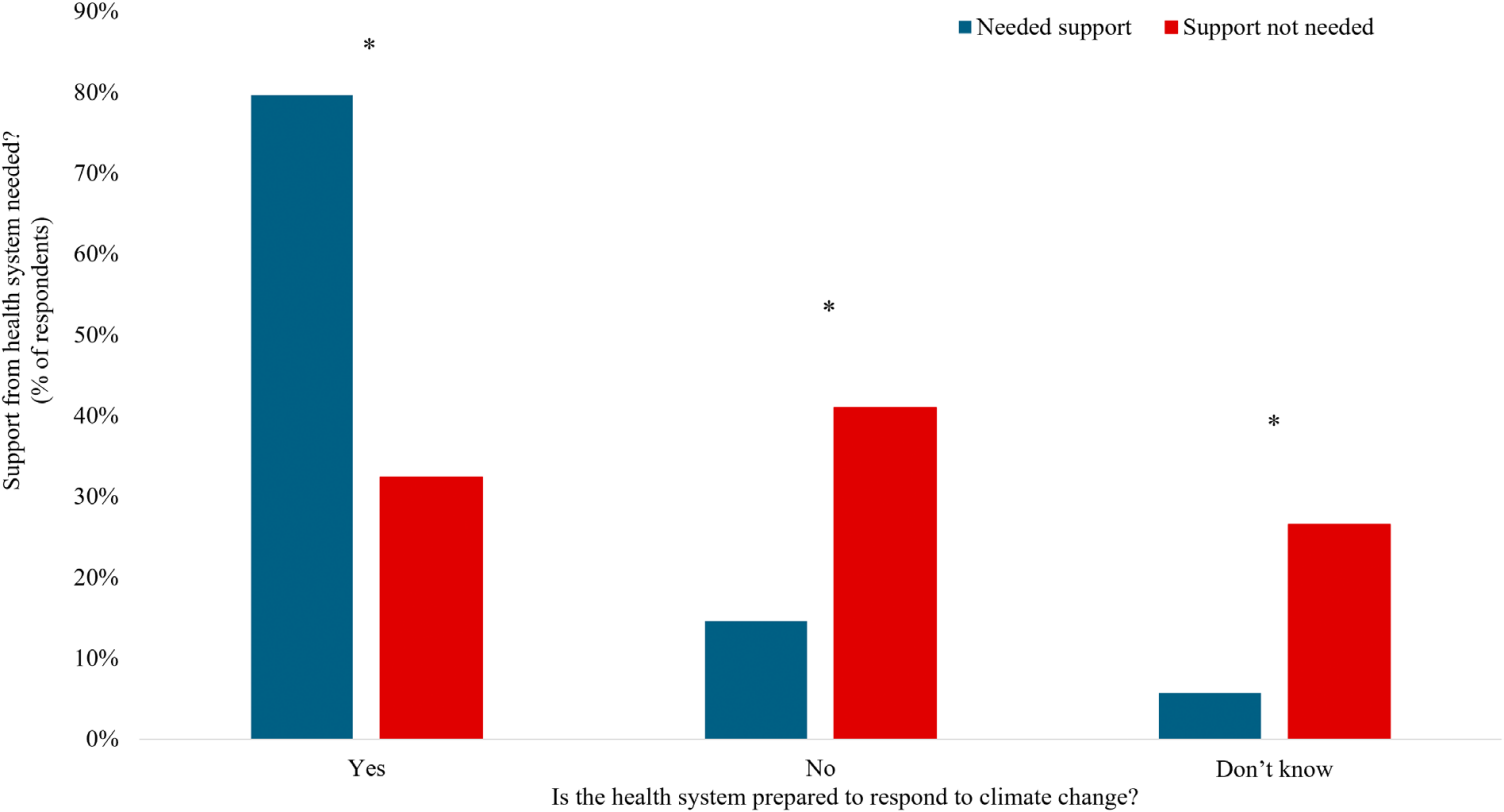
Effects of requiring support from the health system due to a climate‐related event on perceptions of health system preparedness. * indicate significant differences at *p* < 0.05 level, *n* = 3526.

Only 20% (1213) of respondents thought that the health system needed more capacity to respond to climate‐related events. Experience of needing care following a climate‐related event significantly influenced opinions of health system preparedness (*χ* ^2^(2) = 577.30; *p* < 0.001). Of the people who needed medical or psychological support because of a climate‐related event (821/3632, 23%), a higher proportion (80%, 654/821) were confident in the health system's ability to respond to climate‐related emergencies compared with those who did not need attention (32%, *n* = 889/2741; *z* = 13.96, *p* < 0.001) and were less likely to say ‘No’ (15% vs. 41%; *z* = −13.91, *p* < 0.001) or ‘Don't know’ (6% vs. 27%; *z* = −12.69, *p* < 0.001) to the question of whether the health system is prepared to respond to climate change (Figure 2).

Opinions differed regarding willingness to pay more than the current 2% Medicare levy to strengthen the health system's ability to cope with climate‐related events (*χ* ^2^(2) = 804.31; *p* < 0.001; *n* = 6030). Significantly more Australians were willing to increase the levy (‘Yes’: *n* = 2670, 44% vs. ‘No’: *n* = 2374, 39%: *z* = 4.17, *p* < 0.001). Fewer (986, 16%) were unsure (‘Yes’ vs. ‘Don't know’: *z* = 29.84, *p* < 0.001, ‘No’ vs. ‘Don't know’: *z* = 25.17, *p* < 0.001).

## 4 Discussion

### 4.1 The State of Play

The AU‐CLARITY survey revealed that most surveyed Australians believed that climate change is a global emergency, caused by human activity, and that Australia is feeling its effects. Australians' perceptions reflect similar response patterns in surveys conducted in Australia, the United States, United Kingdom, Canada and Germany [5, 8, 11, 24]. However, over half of the Australians surveyed felt that climate change posed little or no threat to their own health. Perception of personal risk varied based on demographic factors and personal experiences. Groups typically more vulnerable to the health effects of climate change, including women, multilingual individuals and First Nations people, reported more concern about its health risks [10, 25, 26, 27, 28].

Mirroring previous studies, having experienced a climate‐related event (e.g., a bushfire or flood) also greatly increased perception of health risks [6, 9, 11, 27]. Higher education was linked to greater perception of personal health risk, which may be tied to political views or media exposure [8, 10, 27]. Counter‐intuitively, despite elevated threats to regional areas from climate change [27, 29], people in regional or remote areas were less concerned about their personal risk, although individuals with higher educational attainment perceived increased personal risk. One explanation is that people frequently identify other groups (e.g., elderly or very young) or distant groups (e.g., other parts of the country or in developing countries) as being at higher risk [5, 11]. In our survey, personal risk perception reduced with age, despite vulnerability to climate‐related harms increasing with age [7]. This calls for targeted public messaging to help individuals better evaluate their own risk and take action to protect their health [1]. As trusted professionals, clinicians are well placed to provide health consumers with climate change information and contribute to public and policy advocacy efforts (Box 1). Previous research suggests that health professionals view climate change as a public health threat, but barriers may inhibit them from communicating climate‐related health risks (e.g., lack of time, knowledge or support structures) [30].

BOX 1 **Implications for policymakers and clinicians.**

**Climate change messaging—Timely messaging from the right messenger to the right person.**

There has traditionally been a strong focus on reaching climate sceptics. This study shows that there is a significant proportion of people who are uncertain about the effects of climate change on health systems, and the system's capacity to respond to climate change‐related emergencies. Targeting those ‘on the fence’ may help shift public opinion and support for climate change‐related policies.

Receiving advice from one's community or from trusted professionals may also influence opinions. Support for community outreach programmes and training for health professionals can create opportunities to reach sceptical and uncertain people.

Balanced messaging about the costs of climate change to health and healthcare systems, that acknowledges cost of living pressures, may increase willingness to contribute more than the current 2% Medicare levy.

### 4.2 Health System Satisfaction and Confidence in Healthcare's Capacity to Respond

Overall, surveyed Australians were satisfied with their healthcare. Men, older individuals, those who spoke English at home and those with higher educational attainment, were more likely to be satisfied. Many believed that the health system either had the capacity to respond to climate change‐related events, or was not being affected by climate change. Confidence in healthcare's response capacity was not uniform; a third were unsure about the health system's preparedness. Women, older individuals and those with chronic conditions were less confident about the health system's response capacity. These views may reflect common experiences of these groups, including low affordability and accessibility of care both to general practitioners and specialists [18]. Having personal experience of a climate‐related event such as bushfire or flood, and requiring care following the event, increased confidence in health system readiness.

Although our results speak well of Australian healthcare and its professionals, the findings remain concerning; climate change directly impacts health systems through immediate damage during events, and longer term effects, which can exacerbate pre‐existing inequities and access [3]. Additionally, the ever‐present impact of climate change on the health workforce compounds already high rates of burnout and workforce attrition, particularly in rural areas [31]. Mis‐estimation of climate change's effects may reduce support for policies to increase system and workforce resilience.

### 4.3 First Nations Respondents

Contrary to previous research [32, 33], our First Nations respondents were more confident about health system preparedness than First Nations respondents in other research, with slightly higher confidence among those residing in metropolitan areas compared with those in the regional/remote areas. Satisfaction with healthcare was high for First Nations respondents. Previous experience with the health system has been shown to inform trust and beliefs about healthcare quality [33]; aspects of care for First Nations people have improved over the last decade, including antenatal care, and greater access to services and hospital procedures [34]. Australian research has also shown improvement in hospital care experiences among people identifying as First Nations [35], which may partially account for the satisfaction rate. The survey enrolled more male and metropolitan residents who identified as First Nations than national averages [36] with more First Nations respondents reporting being affected by climate change. These factors potentially contributed to the positive perceptions of preparedness.

### 4.4 Health for All

Participant confidence in the health system's ability to respond to climate‐related events increased with prior experience and needing care following an event. Previous research has shown that hospitals often remain functional despite the impacts of consecutive disasters and disruptions [37]. Direct personal experience of receiving high quality care despite encountering a disaster may have positively influenced personal opinions, compared with people without these experiences who may be influenced by media portrayals of health services struggling to cope [38].

Australia recently launched its National Health and Climate Strategy, emphasising Health‐in‐All‐Policies [4, 39]. Our findings reinforce the need to address disparities across demographic groups. Policymakers should prioritise those at greater risk of harm from climate change, including women, those with chronic conditions and multilingual individuals [26, 32]. Another priority is building transparent engagement strategies linking climate science, health risks and system preparedness. There remain sceptical groups: disbelievers, or those with uncertainty about the impacts of climate change. Some groups known to be at higher risk (e.g., older individuals; those in rural areas) were less concerned about climate change's effects on their health, creating multiple challenges for policymakers and health systems. Optimising climate and health messaging for specific populations and increasing training and support for health professionals to engage with patients, particularly those at risk of harm, are needed. Specific climate and health messaging about the true costs to health (e.g., in lost productivity and quality of life) and health systems (e.g., costs to bolster system resilience vs. disaster recovery) may also increase Australians' willingness to fund efforts to improve health system resilience and reduce uncertainty in healthcare's preparedness to respond (Box 1).

### 4.5 Strengths and Limitations

This study used a nationally representative sample and, to our knowledge, was the first survey examining the general public's perceptions of climate change effects on the Australian health system, unlike prior surveys, which focused on climate change health impacts or on efforts to reduce carbon emissions from healthcare [11, 40]. Some items were drawn from international surveys (e.g., Canada, United Kingdom [8, 10]) and from previously published Australian surveys, which facilitated comparisons [18].

As to limitations, there is potential selection bias in using a panel receiving incentives, and variations in the interpretation of questions. Mitigating this, we obtained a representative sample, and results are congruent with other health and climate change surveys [18, 40]. Some statistical analyses (e.g., inclusion of interactions) may contribute to overestimations of associations in odds ratios and affect family‐wise error rates. First Nations respondents were overrepresented compared with the population [36], potentially affecting subgroup analyses. Perceptions on healthcare's readiness to respond to climate change‐related emergencies may not reflect opinions on longer term impacts of climate change on healthcare delivery.

## 5 Conclusion

Australians recognise climate change as a health challenge, but perceptions vary by demographics and prior experiences. There exists public scepticism and uncertainty about the effects of climate change on health systems. To ensure support for climate and health policies, policymakers need to address the factors that drive opinions on climate and health.

## Author Contributions

Jeffrey Braithwaite, Carolynn L. Smith, Louise A. Ellis, Yvonne A. Zurynski and S. Bruce Dowton: conceptualisation. Carolynn L. Smith and Jeffrey Braithwaite: writing – original draft. Carolynn L. Smith: formal analysis. Louise A. Ellis and Kate Churruca: validation, formal analysis. Yvonne A. Zurynski, Louise A. Ellis, Georgia Fisher, Janet C. Long, Lisa Pagano, Samantha Spanos, Christina Rojas and S. Bruce Dowton: writing – review and editing. All authors read and approved the final version of the manuscript.

## Funding

Funding was provided by Macquarie University. The funder played no role in the research design, execution, analysis, interpretation and reporting of the results.

## Disclosure

Not commissioned; externally peer reviewed. Microsoft Co‐pilot (AI) was used to refine the wording in the abstract and to verify the interpretation of the statistical analyses. The article was written and revised by one or more of the co‐authors without the assistance of AI, and reviewed and approved by all authors.

## Conflicts of Interest

The authors declare no conflicts of interest.

## Supporting information


**Table S1:** Checklist for Reporting Results of Internet E‐Surveys (CHERRIES) guidelines for electronic surveys.
**Table S2:** Checklist for Reporting Of Survey Studies (CROSS).
**Table S3:** Health conditions.
**Table S4:** Health services accessed.

## Data Availability

The de‐identified data we analysed are not publicly available, but requests to the corresponding author for the data will be considered on a case‐by‐case basis.
