# Supplementary material for: The AUstralian‐CLimate and Health Attitudes, RIsk and Trust in the sYstem (AU‐CLARITY) Study: A National, Representative Survey
Source: Med J Aust. 2026 Sep 10;224(9):e70293. doi: 10.5694/mja2.70293 (PMC13561666; doi:10.5694/mja2.70293)
Supplement: Supplementary file 1 [file MJA2-224-0-s001.pdf]

Supporting Information

Supplementary material

This appendix was part of the submitted manuscript and has been peer reviewed. It is posted as supplied by the authors.

Appendix to: Braithwaite J, Smith CL, Zurynski YA, et al. The AUstralian-CLimate and Health Attitudes, RIsk and Trust in the sYstem (AU-CLARITY) Study: A national, representative survey. *Med J Aust* 2026; doi: 10.5694/mja2.70293.

Section 1:

Table S1. Checklist for Reporting Results of Internet E-Surveys (CHERRIES) guidelines for electronic surveys

Item Category	*Checklist Item*	*Explanation*	*Page number in manuscript*
**Design**	Describe survey design	Describe target population, sample frame. Is the sample a convenience sample? (In “open” surveys this is most likely.)	4-6
**IRB (Institutional Review Board) approval and informed consent process**	IRB approval	Mention whether the study has been approved by an IRB.	6
	Informed consent	Describe the informed consent process. Where were the participants told the length of time of the survey, which data were stored and where and for how long, who the investigator was, and the purpose of the study?	5
	Data protection	If any personal information was collected or stored, describe what mechanisms were used to protect unauthorized access.	5
**Development and pre-testing**	Development and testing	State how the survey was developed, including whether the usability and technical functionality of the electronic questionnaire had been tested before fielding the questionnaire.	5
**Recruitment process and description of the sample having access to the questionnaire**	Open survey versus closed survey	An “open survey” is a survey open for each visitor of a site, while a closed survey is only open to a sample which the investigator knows (password-protected survey).	4,5
	Contact mode	Indicate whether or not the initial contact with the potential participants was made on the Internet. (Investigators may also send out questionnaires by mail and allow for Web-based data entry.)	5
	Advertising the survey	How/where was the survey announced or advertised? Some examples are offline media (newspapers), or online (mailing lists – If yes, which ones?) or banner ads (Where were these banner ads posted and what did they look like?). It is important to know the wording of the announcement as it will heavily influence who chooses to participate. Ideally the survey announcement should be published as an appendix.	4,5
**Survey administration**	Web/E-mail	State the type of e-survey (eg, one posted on a Web site, or one sent out through e-mail). If it is an e-mail survey, were the responses entered manually into a database, or was there an automatic method for capturing responses?	4,5
	Context	Describe the Web site (for mailing list/newsgroup) in which the survey was posted. What is the Web site about, who is visiting it, what are visitors normally looking for? Discuss to what degree the content of the Web site could pre-select the sample or influence the results. For example, a survey about vaccination on a anti-immunization Web site will have different results from a Web survey conducted on a government Web site	4,5
	Mandatory/voluntary	Was it a mandatory survey to be filled in by every visitor who wanted to enter the Web site, or was it a voluntary survey?	4,5
	Incentives	Were any incentives offered (eg, monetary, prizes, or non-monetary incentives such as an offer to provide the survey results)?	4,5
	Time/Date	In what timeframe were the data collected?	4,5
	Randomization of items or questionnaires	To prevent biases items can be randomized or alternated.	4,5
	Adaptive questioning	Use adaptive questioning (certain items, or only conditionally displayed based on responses to other items) to reduce number and complexity of the questions.	4,5
	Number of Items	What was the number of questionnaire items per page? The number of items is an important factor for the completion rate.	See below
	Number of screens (pages)	Over how many pages was the questionnaire distributed? The number of items is an important factor for the completion rate.	See below
	Completeness check	It is technically possible to do consistency or completeness checks before the questionnaire is submitted. Was this done, and if “yes”, how (usually JAVAScript)? An alternative is to check for completeness after the questionnaire has been submitted (and highlight mandatory items). If this has been done, it should be reported. All items should provide a non-response option such as “not applicable” or “rather not say”, and selection of one response option should be enforced.	See below
	Review step	State whether respondents were able to review and change their answers (eg, through a Back button or a Review step which displays a summary of the responses and asks the respondents if they are correct).	See below
**Response rates**	Unique site visitor	If you provide view rates or participation rates, you need to define how you determined a unique visitor. There are different techniques available, based on IP addresses or cookies or both.	4,5
	View rate (Ratio of	Requires counting unique visitors to the first page of the	4,5

	unique survey visitors/unique site visitors)	survey, divided by the number of unique site visitors (not page views!). It is not unusual to have view rates of less than 0.1 % if the survey is voluntary.	
	Participation rate (Ratio of unique visitors who agreed to participate/unique first survey page visitors)	Count the unique number of people who filled in the first survey page (or agreed to participate, for example by checking a checkbox), divided by visitors who visit the first page of the survey (or the informed consents page, if present). This can also be called "recruitment" rate.	4,5
	Completion rate (Ratio of users who finished the survey/users who agreed to participate)	The number of people submitting the last questionnaire page, divided by the number of people who agreed to participate (or submitted the first survey page). This is only relevant if there is a separate "informed consent" page or if the survey goes over several pages. This is a measure for attrition. Note that "completion" can involve leaving questionnaire items blank. This is not a measure for how completely questionnaires were filled in. (If you need a measure for this, use the word "completeness rate".)	4,5
**Preventing multiple entries from the same individual**	Cookies used	Indicate whether cookies were used to assign a unique user identifier to each client computer. If so, mention the page on which the cookie was set and read, and how long the cookie was valid. Were duplicate entries avoided by preventing users access to the survey twice; or were duplicate database entries having the same user ID eliminated before analysis? In the latter case, which entries were kept for analysis (eg, the first entry or the most recent)?	4,5
	IP check	Indicate whether the IP address of the client computer was used to identify potential duplicate entries from the same user. If so, mention the period of time for which no two entries from the same IP address were allowed (eg, 24 hours). Were duplicate entries avoided by preventing users with the same IP address access to the survey twice; or were duplicate database entries having the same IP address within a given period of time eliminated before analysis? If the latter, which entries were kept for analysis (eg, the first entry or the most recent)?	4,5
	Log file analysis	Indicate whether other techniques to analyze the log file for identification of multiple entries were used. If so, please describe.	4,5
	Registration	In "closed" (non-open) surveys, users need to login first and it is easier to prevent duplicate entries from the same user. Describe how this was done. For example, was the survey never displayed a second time once the user had filled it in, or was the username stored together with the survey results and later eliminated? If the latter, which entries were kept for analysis (eg, the first entry or the most recent)?	4,5
**Analysis**	Handling of incomplete questionnaires	Were only completed questionnaires analysed? Were questionnaires which terminated early (where, for example, users did not go through all questionnaire pages) also analysed?	5,6
	Questionnaires submitted with an atypical timestamp	Some investigators may measure the time people needed to fill in a questionnaire and exclude questionnaires that were submitted too soon. Specify the timeframe that was used as a cut-off point, and describe how this point was determined.	See below
	Statistical correction	Indicate whether any methods such as weighting of items or propensity scores have been used to adjust for the non-representative sample; if so, please describe the methods.	5,6

Checklist for Reporting Results of Internet E-Surveys (CHERRIES) details

**Checklist item**	**Explanation**	**Response**
Number of Item	What was the number of questionnaire items per page?<br>The number of items is an important factor for the completion rate	5-6 items
Number of screens (pages)	Over how many pages was the questionnaire distributed? The number of items is an important factor for the completion rate	9 pages
Completeness check	It is technically possible to do consistency or completeness checks before the questionnaire is submitted. Was this done, and if "yes", how (usually JAVAScript)? An alternative is to check for completeness after the questionnaire has been submitted (and highlight mandatory items). If this has been done, it should be reported. All items should provide a non-response option such as "not applicable" or "rather not say", and selection of one response option should be enforced.	Dynata conducted a completeness check prior to providing the deidentified data.
Review step	State whether respondents were able to review and change their answers (eg, through a Back button or a Review step which displays a summary of the responses and asks the respondents if they are correct)	Respondents were able to use the 'back' button to review or change responses prior to submission
Questionnaires submitted with an atypical timestamp	Some investigators may measure the time people needed to fill in a questionnaire and exclude questionnaires that were submitted too soon. Specify the timeframe that was used as a cut-off point, and describe how this point was determined.	Timestamps were reviewed against completeness. No submissions were excluded based on timestamp

Table S2. Checklist for Reporting Of Survey Studies (CROSS)

Section/topic	*Item*	*Item description*	*Reported on page #*
		**Title and abstract**	
Title and abstract	1a	State the word "survey" along with a commonly used term in title or abstract to introduce the study's design.	1,2
	1b	Provide an informative summary in the abstract, covering background, objectives, methods, findings/results, interpretation/discussion, and conclusions.	2
		**Introduction**	
Background	2	Provide a background about the rationale of study, what has been previously done, and why this survey is needed.	4
Purpose/aim	3	Identify specific purposes, aims, goals, or objectives of the study.	4
		**Methods**	
Study design	4	Specify the study design in the methods section with a commonly used term (e.g., cross-sectional or longitudinal).	2, 4-6
Data collection methods	5a	Describe the questionnaire (e.g., number of sections, number of questions, number and names of instruments used).	4-6
	5b	Describe all questionnaire instruments that were used in the survey to measure concepts. Report target population, reported validity and reliability information, scoring/classification procedure, and reference links (if any).	4-6
	5c	Provide information on pretesting of the questionnaire, if performed (in the article or in an online supplement). Report the method of pretesting, number of times questionnaire was pre-tested, number and demographics of participants used for pretesting, and the level of similarity of demographics between pre-testing participants and sample population.	4-6
	5d	Questionnaire if possible, should be fully provided (in the article, or as appendices or as an online supplement).	Online supplement
Sample characteristics	6a	Describe the study population (i.e., background, locations, eligibility criteria for participant inclusion in survey, exclusion criteria).	6, 18
	6b	Describe the sampling techniques used (e.g., single stage or multistage sampling, simple random sampling, stratified sampling, cluster sampling, convenience sampling). Specify the locations of sample participants whenever clustered sampling was applied.	4,5
	6c	Provide information on sample size, along with details of sample size calculation.	18
	6d	Describe how representative the sample is of the study population (or target population if possible), particularly for population-based surveys.	6
Survey administration	7a	Provide information on modes of questionnaire administration, including the type and number of contacts, the location where the survey was conducted (e.g., outpatient room or by use of online tools, such as SurveyMonkey).	4,5
	7b	Provide information of survey's time frame, such as periods of recruitment, exposure, and follow-up days.	4,5
	7c	Provide information on the entry process:<br>–>For non-web-based surveys, provide approaches to minimize human error in data entry.<br>–>For web-based surveys, provide approaches to prevent "multiple participation" of participants.	5
Study preparation	8	Describe any preparation process before conducting the survey (e.g., interviewers' training process, advertising the survey).	4,5
Ethical considerations	9a	Provide information on ethical approval for the survey if obtained, including informed consent, institutional review board [IRB] approval, Helsinki declaration, and good clinical practice [GCP] declaration (as appropriate).	6
	9b	Provide information about survey anonymity and confidentiality and describe what mechanisms were used to protect unauthorized access.	5,6
Statistical analysis	10a	Describe statistical methods and analytical approach. Report the statistical software that was used for data analysis.	5,6
	10b	Report any modification of variables used in the analysis, along with reference (if available).	5,6
	10c	Report details about how missing data was handled. Include rate of missing items, missing data mechanism (i.e., missing completely at random [MCAR], missing at random [MAR] or missing not at random [MNAR]) and methods used to deal with missing data (e.g., multiple imputation).	5,6
	10d	State how non-response error was addressed.	5,6
	10e	For longitudinal surveys, state how loss to follow-up was addressed.	N/A
	10f	Indicate whether any methods such as weighting of items or propensity scores have been used to adjust for non-representativeness of the sample.	6
	10g	Describe any sensitivity analysis conducted.	NA
		**Results**	
Respondent characteristics	11a	Report numbers of individuals at each stage of the study. Consider using a flow diagram, if possible.	4,5
	11b	Provide reasons for non-participation at each stage, if possible.	4,5
	11c	Report response rate, present the definition of response rate or the formula used to calculate response rate.	5
	11d	Provide information to define how unique visitors are determined. Report number of unique visitors along with relevant proportions (e.g., view proportion, participation proportion, completion proportion).	5
Descriptive results	12	Provide characteristics of study participants, as well as information on potential confounders and assessed outcomes.	4,6,18
Main findings	13a	Give unadjusted estimates and, if applicable, confounder-adjusted estimates along with 95% confidence intervals and p-values.	6-9,18-20
	13b	For multivariable analysis, provide information on the model building process, model fit statistics, and model assumptions (as appropriate).	5,6
	13c	Provide details about any sensitivity analysis performed. If there are considerable amount of missing data, report sensitivity analyses comparing the results of complete cases with that of the imputed dataset (if	N/A

		possible).	
**Discussion**			
Limitations	14	Discuss the limitations of the study, considering sources of potential biases and imprecisions, such as non-representativeness of sample, study design, important uncontrolled confounders.	12,13
Interpretations	15	Give a cautious overall interpretation of results, based on potential biases and imprecisions and suggest areas for future research.	12,13
Generalizability	16	Discuss the external validity of the results.	12,13
**Other sections**			
Role of funding source	17	State whether any funding organization has had any roles in the survey's design, implementation, and analysis.	14
Conflict of interest	18	Declare any potential conflict of interest.	14
Acknowledgements	19	Provide names of organizations/persons that are acknowledged along with their contribution to the research.	14

N/A = not applicable

Table S3. Health conditions

Condition	n	%†
Mental health conditions (such as depression or anxiety)	1317	(22)
Back problems	1098	(18)
Arthritis	922	(15)
Asthma	766	(13)
Diabetes	499	(8)
Other (please specify)*	353	(6)
Osteoporosis	292	(5)
Cardiovascular disease (such as coronary heart disease or stroke)	244	(4)
Endometriosis	186	(3)
Chronic obstructive pulmonary disease	153	(3)
Cancer (such as lung or colorectal cancer)	142	(2)
Kidney disease	93	(2)
Dementia	37	(1)

† Multiple response question, therefore percent is > 100.

* Other long-term health conditions not included in the Australian Institute of Health and Welfare list.

Table S4. Health services accessed

Health service	n	%†
General practice (GP)	4197	(70)
Pharmacist	3357	(56)
Dentist or dental services	2492	(41)
Public hospital	2243	(37)
Specialist doctor outside hospital (e.g. a cardiologist, surgeon, psychiatrist)	1306	(22)
Private hospital	1017	(17)
Allied health service provider (e.g., a physiotherapist, dietician, etc.)	954	(16)
Nurse who works in a general practice	900	(15)
Counsellor or psychologist	746	(12)
None of these*	434	(7)
Alternative therapies practitioner (e.g., acupuncture, naturopathy, etc.)	308	(5)
Community-based healthcare service (excluding an Aboriginal Community Controlled Health Service)	241	(4)
Specialist pain clinic	233	(4)
Aboriginal Community Controlled Health Service	93	(2)
An Indigenous health practitioner	24	(0)

† Multiple response question, therefore % is > 100.
* No further details were collected on those who use a service that was not listed.

Section 2:

AU-CLARITY Survey questions

Question text	Sub questions	Answer options/ format
Consent to complete survey	*Show PICF form*	Yes/No
What is your gender?		*[Select one]:*<br>- Male<br>- Female<br>- Non-binary<br>- Prefer not to answer<br>- Other
What is your gender?		*[text box for typing response if 'Other' selected]*
In what year were you born? (enter 4-digit birth year; for example, 1976)		Open ended four digit numerical
What is your post code? (enter a 4-digit postcode; for example, 2000)		Open ended four digit numerical
Are you of Aboriginal and/or Torres Strait Islander origin?		- No<br>- Aboriginal origin<br>- Torres Strait Islander origin<br>- Both Aboriginal and Torres Strait Islander origin
Do you speak a language other than English at home?		- Yes<br>- No
What is the highest level of education that you have completed? If you are currently studying, but have not completed a qualification, please answer with the highest level you have completed.		*[Select one]:*<br>- Postgraduate Degree<br>- Graduate Diploma / Graduate Certificate<br>- Bachelor's Degree<br>- Advanced Diploma / Diploma<br>- Certificate level<br>- Secondary school<br>- Other (please specify)<br>- Have not completed any education
What is the highest level of education that you have completed? If you are currently studying, but have not completed a qualification, please answer with the highest level you have completed.		*[text box for typing response if 'Other' selected]*
What is your household income per week before tax?		*[Select one]:*<br>- Negative income<br>- Nil income<br>- $1-$299 per week<br>- $300-$599 per week<br>- $600-$999 per week<br>- $1,000-$1,999 per week<br>- $2,000-$2,999 per week<br>- $3,000-$3,999 per week<br>- $4,000-$4,999 per week<br>- $5,000-$5,999 per week<br>- $6,000-$7,999 per week<br>- $8,000 or more per week<br>- Prefer not to say
What is your current work status?		*[Select one]:*<br>- Full-time paid work (>35 hours per week)<br>- Part-time paid work (<35 hours per week)<br>- Unemployed<br>- Retired<br>- Other
What is your current work status?		*[text box for typing response if 'Other' selected]*
Who currently lives with you in your household?		*[Select all that apply]*<br>- *Alone*<br>- *Spouse/Partner*<br>- *Children*<br>- *Parents*<br>- *Siblings*<br>- *Other relatives*<br>- *Friends/housemates*<br>- *Other (please specify)*
Are you currently a:		*[Select one]*<br>- Full-time student

AU-CLARITY Survey questions

Question text	Sub questions	Answer options/ format
		- Part-time student<br>- Neither of these
Do you undertake any unpaid work or carer roles (e.g., volunteer, carer, household duties?)		*[select one]*<br>- Yes<br>- No
Do you undertake any unpaid work or carer roles?		*[select all that apply]*<br>- Full-time household duties<br>- Part time household duties<br>- Full-time carer<br>- Part-time carer<br>- Voluntary work<br>- Other (please specify)
Do you undertake any unpaid work or carer roles?		*[text box for typing response if 'Other' selected]*
In the **last 12 months**, did any of the following happen to you because of a shortage of money?		*[Select as many as apply]:*<br>- Could not pay the electricity, gas or telephone bills on time<br>- Could not pay the mortgage or rent on time<br>- Pawned or sold something<br>- Went without meals<br>- Was unable to heat home<br>- Asked for financial help from friends or family<br>- Asked for help from welfare/community organisations<br>- Could not pay for health care or medicine you needed<br>- None of these
Do you have a Medicare Card?		*[Select one]:*<br>- Yes<br>- No
Do you have private health insurance?		*[Select one]:*<br>- Yes<br>- No<br>- Don't know
In general, how would you describe your own health?		*[Select one]:*<br>- Excellent<br>- Very good<br>- Good<br>- Fair<br>- Poor
At the moment, do you have any of the chronic illnesses listed, that have lasted, or are likely to last, for six months or more? If so, please select which ones		*[Select as many as apply]*<br>- Arthritis<br>- Asthma<br>- Back problems<br>- Cancer (such as lung or colorectal cancer)<br>- Cardiovascular disease (such as coronary heart disease or stroke)<br>- Chronic obstructive pulmonary disease<br>- Dementia<br>- Diabetes<br>- Kidney disease<br>- Endometriosis<br>- Mental health conditions (such as depression or anxiety)<br>- Osteoporosis<br>- No, none of the above<br>- Other (please specify)
At the moment, do you have any of the chronic illnesses listed, that have lasted, or are likely to last, for six months or more? If so, please select which ones		*[text box for typing response if 'Other' selected]*
Please indicate which of the following health services you have accessed **in person** in the **last 12 months**:		*[Select as many as apply]*<br>- A public hospital<br>- A private hospital<br>- A GP<br>- A nurse who works in a general practice<br>- A dentist or dental services

AU-CLARITY Survey questions

Question text	Sub questions	Answer options/ format
		- A pharmacist<br>- A specialist doctor outside hospital (e.g. a cardiologist, surgeon, psychiatrist)<br>- A specialist pain clinic<br>- A counsellor or psychologist<br>- A community-based healthcare service (excluding an Aboriginal Community Controlled Health Service)<br>- An Aboriginal Community Controlled Health Service<br>- An allied health service provider (e.g., a physiotherapist, dietician, etc.)<br>- An alternative therapies practitioner (e.g., acupuncture, naturopathy, etc.)<br>- An indigenous health practitioner<br>- None of these
How satisfied were you with your most recent experience with the service?	- A public hospital<br>- A private hospital<br>- A GP<br>- A nurse who works in a general practice<br>- A dentist or dental services<br>- A pharmacist<br>- A specialist doctor outside hospital (e.g., a cardiologist, surgeon, psychiatrist)<br>- A specialist pain clinic<br>- A counsellor or psychologist<br>- A community-based healthcare service (excluding an Aboriginal Community Controlled Health Service)<br>- An Aboriginal Community Controlled Health Service<br>- An allied health service provider, such as a physiotherapist or dietician<br>- An alternative therapies practitioner e.g. acupuncture, naturopathy etc<br>- An Indigenous health practitioner	*[Select one]*<br>- Very satisfied<br>- Somewhat satisfied<br>- Neither satisfied nor dissatisfied<br>- Somewhat dissatisfied<br>- Very dissatisfied<br>- N/A
Thinking about **<u>all</u>** instances of healthcare you have received over the last 12 months, how satisfied were you with the quality of care that you received?		*[Select one]*<br>- Very satisfied<br>- Somewhat satisfied<br>- Neither satisfied nor dissatisfied<br>- Somewhat dissatisfied<br>- Very dissatisfied<br>- Haven't received healthcare in the last 12 months<br>- Don't know
The following questions ask about how you have been feeling **during the past 30 days**. For each question, please select the number that best describes how often you had this feeling.	- …nervous?<br>- …hopeless?<br>- …restless or fidgety?<br>- …so depressed that nothing could cheer you up?<br>- …that everything was an effort?<br>- …worthless?	*[Select one]*<br>- *All of the time*<br>- *Most of the time*<br>- *Some of the time*<br>- *A little of the time*<br>- *None of the time*
In general, how concerned are you, if at all, about climate change?		*[Select one]*<br>- Very concerned<br>- Fairly concerned<br>- Not very concerned<br>- Not at all concerned<br>- Don't know
To what extent do you agree or disagree with each of the following statements?	- Climate change is caused by human activity<br>- Climate change is a global emergency<br>- The world's climate is already changing<br>- Australia is already feeling the effects of climate change	*[Select one]*<br>- Strongly agree<br>- Tend to agree<br>- Neither agree nor disagree<br>- Tend to disagree<br>- Strongly disagree<br>- Don't know

AU-CLARITY Survey questions

Question text	Sub questions	Answer options/ format
How much of a threat, if at all, do you think climate change poses to each of the following?	- Your health personally<br>- Your family's health<br>- The health of Australians<br>- The health of people globally	*[Select one]*<br>- A great deal of threat<br>- A fair amount of threat<br>- Not very much threat<br>- No threat at all<br>- Don't know
How concerned or not concerned are you about the following climate risks/impacts on yours or your family's health (where 0 is not at all concerned and 10 is very concerned)?	- Current threats to your health and well-being because of climate impacts<br>- Increased number of very hot days and associated heat waves in the future affecting your health<br>- Impacts to the health of future generations	[*Numeric rating scale*]<br>(0 is not concerned at all and 10 is very concerned) + unsure
How concerned are you about the following health risks/impacts from climate change (where 0 is not concerned at all and 10 is very concerned)? [RANDOMIZE]	- Heat stroke<br>- Water quality and availability<br>- Work and recreation outside during extreme heat<br>- Personal climate anxiety<br>- Worry about wellbeing of future generations<br>- Diseases spread in water, such as gastrointestinal illnesses<br>- Cardiorespiratory (heart or lung) problems caused by air pollution<br>- Asthma and allergies<br>- Stress from evacuation during extreme weather events<br>- Bushfire smoke causing respiratory problems<br>- Health impacts of increased drought due to increasing heat<br>- Availability and accessibility of high-quality food<br>- Diseases spread by insects, such as Ross River Virus, Japanese Encephalitis	[*Numeric rating scale*]<br>(0 is not concerned at all and 10 is very concerned) + unsure
Does climate change make you feel any of the following?	- Sad<br>- Helpless<br>- Anxious<br>- Afraid<br>- Optimistic<br>- Angry<br>- Guilty<br>- Ashamed<br>- Hurt<br>- Depressed<br>- Despair<br>- Grief<br>- Powerless<br>- Indifferent	*[Select one]*<br>- Yes<br>- No<br>- Prefer not to say
Have you ever been personally affected by any of the following in the last **five years**? (check all that apply)		*[Select all that apply]*<br>- Bushfire<br>- Flood<br>- Drought<br>- Heatwave<br>- Severe storm or cyclone<br>- Extreme cold weather<br>- Infectious disease that has become more common due to climate change (e.g. Japanese encephalitis, Ross River virus, dengue fever)<br>- Other climate-related event<br>- None<br>- I don't know
On a scale of 1 to 5, how would you rate the impact of the recent climate-related event on your physical health?		*[Select one]*<br>- 1 - No impact<br>- 2 - Minor impact<br>- 3 - Moderate impact<br>- 4 - Significant impact

AU-CLARITY Survey questions

Question text	Sub questions	Answer options/ format
		- 5 - Severe impact
On a scale of 1 to 5, how would you rate the impact of the recent climate-related event on your mental health?		*[Select one]*<br>- 1 - No impact<br>- 2 - Minor impact<br>- 3 - Moderate impact<br>- 4 - Significant impact<br>- 5 - Severe impact
Did you need medical attention or support from a health professional, including mental health support, because of the this?		*[Select one]*<br>- Yes<br>- No<br>- Prefer not to say
What health service did you use (e.g., hospital, GP, psychologist, psychiatrist, allied health professional)?		*[Free text response]*
How concerned are you about being impacted by any of the following within the next **five years**?	- Bushfire<br>- Flood<br>- Drought<br>- Heatwave<br>- Severe storm or cyclone<br>- Severe cold<br>- Infectious disease that has become more common due to climate change (e.g. Japanese encephalitis, Ross River virus, dengue fever)<br>- Other climate-related event<br>- Pandemic (a highly contagious disease outbreak, such as COVID-19)	*[Select one]*<br>- Very concerned<br>- Fairly concerned<br>- Not very concerned<br>- Not at all concerned<br>- Don't know
Have you discussed with your doctor how climate-related events, such as heatwaves or smoke from bushfires, may affect your or your family's health?		*[Select one]*<br>- Yes<br>- No
If you are taking any medications, have you discussed with your doctor how exposure to climate-related events (such as heatwaves or bushfire smoke) may interact with your medications?		*[Select one]*<br>- Yes<br>- No
Thinking about emergencies that may affect you within the **next five years,** how confident are you in dealing with these events should they occur?	- Bushfire<br>- Flood<br>- Drought<br>- Heatwave<br>- Severe storm or cyclone<br>- Severe cold<br>- Infectious disease that has become more common due to climate change (e.g. Japanese encephalitis, Ross River virus, dengue fever)<br>- Pandemic (a highly contagious disease outbreak, such as COVID-19)	*[Select one]*<br>- Very confident<br>- Fairly confident<br>- Not very confident<br>- Not at all confident<br>- Don't know
Thinking about the emergencies that may affect you within the **next five years**, do you have an emergency plan that could guide you through these events should they occur?		*[Select one]*<br>- Yes<br>- No
What motivated you to make an emergency plan?		*[Select one]*<br>- I am confident that I can actually do something to help myself

AU-CLARITY Survey questions

Question text	Sub questions	Answer options/ format
(Select the most appropriate option)		- By making an emergency plan, I am improving my or my family's chances of surviving during an emergency<br>- By making an emergency plan, I could reduce the damage to my property during an emergency,<br>- My family or friends encouraged me make an emergency plan<br>- My job, school or community encouraged me to have an emergency plan,<br>- Disasters in other places made me think about making an emergency plan,<br>- Disasters I have experienced made me think about making an emergency plan<br>- Other
What are your main reasons for not having an emergency plan?		*[Free text response]*
Have you (or anyone in your household) taken any of the following actions to prepare for an emergency in the past one year?	SET A<br>- Keeping an updated emergency contact list<br>- Copies of prescriptions in a waterproof container or as an electronic version<br>- Developing an evacuation plan for where to go if you decide to leave and how to get there<br>- Arranging a family meeting place or reconnection plan<br>- Stocking your house with emergency supplies such as 3-day supply of food and drinking water, a first aid kit, a flashlight, a battery-powered radio, extra batteries, personal hygiene items, food for pet or assistance animals<br>- Getting and reviewing property, vehicle and/or life insurance policies<br>- Making a backup plan for support workers/personal assistants and essential suppliers/agencies<br>- Working out how to get timely emergency information and warnings<br>- Getting a home generator<br><br>SET B<br>- Writing down your medical information, such as identification, medications, food and drug allergies, health records etc.<br>- Safely storing important documents (e.g., will, power of attorney, passport, banking information, etc.)<br>- Making arrangements for short-notice/emergency replacement care<br>- Reducing home/property damage by either keeping gutter and downpipes clear of leaves and litter, trimming trees and cutting back overhanging branches, clearing an area around the house, keeping the roof in good condition, installing and maintaining smoke alarm etc.<br>- Putting together an easy-to-carry go-bag with critical items, such as mobile phone, medications, flashlight,	*[Select one]*<br>- *Yes*<br>- No<br>- Partially<br>- Not applicable

AU-CLARITY Survey questions

Question text	Sub questions	Answer options/ format
	extra cash, spare clothing, food and water, blankets, etc.<br>- Speaking with family and/or personal assistant about what to do during an emergency situation<br>- Knowing the manual operations for your medical equipment or assistive technology<br>- Participating in an evacuation drill in the past 12 months<br>- Maintaining at least a quarter tank of petrol (or maintaining adequate electrical charge) in your vehicle at all times	
What communication mediums/channels would you use to get timely information about emergencies or disaster warnings?<br>(Can choose more than one)		*[Select all that apply]*<br>- TV<br>- Radio, such as ABC emergency<br>- Newspaper<br>- Websites of local council or emergency services<br>- Email alert<br>- Voice or text alert to mobile phones<br>- Online news<br>- Smartphone or tablet applications, such as Fires Near Me<br>- Call-in number for updates<br>- Social media, such as X (Twitter), Facebook, Instagram, TikTok<br>- 106 TTY Services (hearing impaired service)<br>- Warning signs in public areas<br>- Other
Have you discussed with your doctor or another health professional your plans for staying healthy and safe in the case of emergency events?		*[Select one]*<br>- Yes<br>- No<br>- Not sure
Which of the following emergency events have you discussed with your doctor or health professional?		*[select all that apply]*<br>- Bushfire<br>- Flood<br>- Drought<br>- Heatwave<br>- Severe storm or cyclone<br>- Extreme cold weather<br>- Infectious disease that has become more common due to climate change (e.g. Japanese encephalitis, Ross River virus, dengue fever)<br>- Other climate-related event<br>- None<br>- I don't know
Which of the following supplies do you currently have on hand in the case of emergency or evacuation?		*[Select all that apply]*<br><br>1. First-aid kit<br>2. Working flashlight<br>3. Working battery-powered portable radio<br>4. Spare batteries<br>5. Mess kit or paper cups, plates, plastic utensils<br>6. Cash or traveller's checks<br>7. Nonelectric can opener or utility knife<br>8. Antibacterial wipes or gel<br>9. Personal hygiene items<br>10. Toilet paper or towelettes<br>11. One change of clothes per person<br>12. Blanket or sleeping bag per person<br>13. Essential medications and medical equipment (puffers, auto-injector)<br>14. Spare glasses or contact lenses<br>15. Hearing aids and batteries<br>16. Extra keys for house and car<br>17. List of emergency phone numbers

AU-CLARITY Survey questions

Question text	Sub questions	Answer options/ format
		18. Mobile phone and charged power pack<br>19. Personal identification items (license, passport, photo ID card, Medicare card)<br>20. Medical alert bracelet<br>21. Bottled water<br>22. Non-perishable food
In the case of an emergency event, how much would you expect to rely on the following for assistance?	- Household members<br>- Family members who do not live with me<br>- Neighbours<br>- Personal care/support worker(s)<br>- State Emergency Service (SES)<br>- Fire services/Rural Fire Service/Fire and Rescue<br>- Police<br>- Emergency relief/recovery organisations such as the Red Cross or the Salvation Army<br>- Local council<br>- My faith communities<br>-	*[Numerical rating scale]*<br>1 (do not expect to reply on at all) to 5 (expect to rely on a great deal) + Not applicable
Do you have a 3 to 7-day stockpile of medications?		*[Select one]:*<br>- Yes<br>- No<br>- I do not take medications
Which of the following statements comes closest to expressing your overall view of the health care system in Australia?		*[Select one]:*<br>- On the whole, the system works pretty well and only minor changes are needed to make it work better; or<br>- There are some good things in our health care system, but fundamental changes are needed to make it work better; or<br>- Our health care system has so much wrong with it that we need to completely rebuild it
Do you believe that the Australian Health System is being affected by climate change?		*[Select one]*<br>- *Yes*<br>- *No*<br>- *Don't know*
Do you believe that the Australian Health System is ready to respond to climate change-related emergencies?		*[Select one]*<br>- *Yes*<br>- *No*<br>- *Don't know*
Do you believe that the Australian Health System contributes to climate change (e.g., through emission of greenhouse gases)?		*[Select one]*<br>- *Yes*<br>- *No*<br>- *Don't know*
Would you be prepared to contribute more than the current 2% Medicare levy on your taxable income?	- If this would strengthen the health systems' ability to cope with climate-related events (e.g., bushfires, floods)?<br>- if this would enable health systems to reduce their carbon footprint?	*[Select one]:*<br>- Yes<br>- No<br>- Don't know
Which of the following areas of the health system do you think need improvement?		*[Select all that apply]:*<br>- Getting better access to care<br>- Getting better quality of care<br>- The cost of care or medicines<br>- More doctors, nurses and other health workers<br>- Listening to patients more<br>- Transparency on health care e.g. different medicines for the same condition, different doctors doing the same procedure, cost of medications, outcomes of treatment<br>- Health professionals' knowledge

AU-CLARITY Survey questions

Question text	Sub questions	Answer options/ format
		- Communication among health professionals who look after me<br>- Use of shared electronic records (such as MyHealth Record)<br>- Better ability to respond to climate-related events (e.g., heatwaves, bushfires, floods)<br>- Lower carbon emissions or waste<br>- No changes are needed<br>- Other (please specify)
Which of the following other areas of the health system do you think need improvement?		*[text box for answer]*
To what extent do you agree or disagree with each of the following statements:	- *The Australian health system is contributing to climate change.*<br><br>- *The Australian health system has a responsibility to reduce its impact on climate change.*<br><br>- *The Australian health system should make reducing its impact on climate change one of its top priorities.*	*[Select one]*<br>- Strongly disagree<br>- Tend to agree<br>- Neither agree nor disagree<br>- Tend to disagree<br>- Strongly agree
To what extent do you support or oppose each of the following measures?	- *Providing more environmentally friendly food in hospitals (for example, more vegetarian, vegan, and locally sourced and seasonal food), even if this means there are fewer options available (for example, less red meat).*<br><br>- *The Australian health system switching its fleet of vehicles (such as ambulances) to electric and hydrogen-power now, even if taxes have to increase to pay for this.*<br><br>- *Building new hospitals to net zero standards, even if taxes have to increase to pay for this*<br><br>- *Considering the environmental impact of treatments when offering them to patients, even if this means people are encouraged to take treatments or medicines that they are less familiar with. For example, people with asthma using powder inhalers, which are more environmentally friendly (but are equally effective).*<br><br>- *The Australian health system reducing waste, for example, by reusing medical equipment (such as blades used for surgeries, which are currently single-use) which has been sterilised, even if this means that you have to use equipment other people have used first.*<br><br>- *GP practices and hospitals increasing the amount of space for public transport (for example, increasing the number of bus stops in front of hospitals) and bicycles, even if this means*	*[Select one]*<br>- Support,<br>- Oppose<br>- Don't know

AU-CLARITY Survey questions

Question text	Sub questions	Answer options/ format
	reducing the amount of available parking for private vehicles.<br><br>- *Require companies that sell to the Australian health system to report the carbon footprint of their products (e.g., gowns, medical devices, pharmaceuticals)*<br><br>- *Funding research to develop more sustainable health systems*	
Please tell us why you support or oppose any of these suggestions	-	*[Free text]*

Thank you very much for your time and comments. That is the end of the survey.

If participating in this survey raised any concerns about how you have been feeling, and you would like to talk to someone, there are a number of support options available to assist you.

You can visit your regular GP, or if you have seen a health professional before to help with problems, you may wish to make an appointment to see them again.

For immediate support, contact Lifeline on 13 11 14, available 24/7, or online text chat from 7pm-4am (AEST) at: lifeline.org.au/get-help/onlineservices/crisis-chat

The beyondblue Support Service also provides free, immediate, short-term counselling advice and referrals to people in Australia via telephone and email 24/7, and web chat from 3pm to midnight (AEST), every day. The Support Service can be contacted on 1300 22 4636 or via email or web chat at: beyondblue.org.au/get-support
